# Implementing an electronic health record dashboard for safe anticoagulant management: learning from qualitative interviews with existing and potential users to develop an implementation process

**DOI:** 10.1186/s43058-022-00262-w

**Published:** 2022-02-02

**Authors:** Geoffrey D. Barnes, Emily Sippola, Allison Ranusch, Linda Takamine, Michael Lanham, Michael Dorsch, Anne Sales, Jeremy Sussman

**Affiliations:** 1 Frankel Cardiovascular Center, Department of Internal Medicine, University of Michigan, 2800 Plymouth Rd, B14 G214, Ann Arbor, MI 48109-2800 USA; 2 Center for Behavioral and Social Sciences in Medicine, University of Michigan, Ann Arbor, USA; 3 Institute for Healthcare Policy and Innovation, University of Michigan, Ann Arbor, USA; 4 Center for Clinical Management Research, Ann Arbor Veterans Health Affairs, Ann Arbor, USA; 5 Department of Learning Health Sciences, University of Michigan, Ann Arbor, USA; 6 College of Pharmacy, University of Michigan, Ann Arbor, USA; 7 Division of General Medicine, Department of Internal Medicine, University of Michigan, Ann Arbor, USA

**Keywords:** Implementation, Population health, Anticoagulation, Pharmacist

## Abstract

**Background:**

Facilitating appropriate care delivery using electronic health record (digital health) tools is increasing. However, frequently used determinants frameworks seldom address key barriers for technology-associated implementation.

**Methods:**

Semi-structured interviews were conducted in two contexts: the national Veterans Health Affairs (VA) following implementation of an electronic dashboard, a population health tool, and the Michigan Anticoagulation Quality Improvement Initiative (MAQI^2^) prior to implementation of a similar electronic dashboard. The dashboard is designed for pharmacist or nurse use to monitor safe outpatient anticoagulant prescribing by physicians and other clinicians We performed rapid qualitative inquiry analysis and selected implementation strategies. Through a stakeholder focus group session, we selected implementation strategies to address determinants and facilitate implementation in the MAQI^2^ sites.

**Results:**

Among 45 interviewees (32 in VA, 13 in MAQI^2^), we identified five key determinants of implementation success: (1) clinician authority and autonomy, (2) clinician self-identity and job satisfaction, (3) documentation and administrative needs, (4) staffing and work schedule, and (5) integration with existing information systems. Key differences between the two contexts included concerns about information technology support and prioritization within MAQI^2^ (prior to implementation) but not VA (after implementation) and concerns about authority and autonomy that differed between the VA (higher baseline levels, more concerns) and MAQI^2^ (lower baseline levels, less concern).

**Conclusions:**

The successful implementation of electronic health record tools requires unique considerations that differ from other types of implementation, must account for the status of implementation, and should address the effects of the tool deployment on clinical staff authority and autonomy. Interviewing both post-implementation and pre-implementation users can provide a robust understanding of implementation determinants.

**Supplementary Information:**

The online version contains supplementary material available at 10.1186/s43058-022-00262-w.

Contributions to the literature

- Implementation efforts that leverage technology-associated tools must address implementation determinants that differ from other types of tools, based on available resources, accuracy, and integration into workflow
- Combining interviews with experiences and pre-implementation sites can help to identify additional determinants of implementation success
- Baseline levels of autonomy significantly impacts how individuals perceive specific barriers to implementing new team-based care delivery processes

## Background

A potential benefit of rapidly growing electronic health record (EHR) use in medicine is the capacity to implement evidence-based, EHR-guided clinical decision support tools, such as the use of best practice alerts (clinical reminders) and population-level dashboards [1–3]. While best practice alerts are typically designed for specific patient-provider interactions, population-level dashboards allow clinicians and/or clinical leaders to survey a large cohort of patients to identify key trends in care delivery. However, few evaluations have addressed the operational and social implementation barriers of these tools for clinical staff. Additionally, how well an electronic tool developed in one clinical setting can be adapted and implemented in an alternative setting remains largely unknown. To better understand these challenges, we set out to study the implementation of a population health dashboard for safe anticoagulant prescribing and monitoring in two distinct settings, one of which was already using the dashboard, the other was not.

All anticoagulant drugs are high-risk/high-benefit medications that are essential for preventing life-threatening complications, such as stroke and other thrombotic conditions [4]. For more than five decades, warfarin was the only available oral anticoagulant in the USA and much of the world. While effective at preventing blood clots, warfarin has a high risk of harm and is burdensome to dose and monitor [4]. Since 2010, oral anticoagulant prescribing has been slowly transitioning from warfarin to newer direct oral anticoagulants (DOACs) [5–8]. This shift has led to a substantial change in who manages anticoagulation prescription changes and how closely patients are followed. Specifically, patients on warfarin are often monitored at least every month to ensure appropriate dosing and drug levels. However, DOAC medications are prescribed using fixed doses without any dosing or drug level monitoring. Therefore, DOAC-treated patients typically do not have frequent interactions with anticoagulation experts, such as nurses and pharmacists working in anticoagulation clinics.

While safer and dramatically simpler to dose and monitor than warfarin, accurate DOAC prescribing is still complicated and adverse events due to incorrect dosing remain relatively common. In fact, multiple studies have identified that as many as 1 in 7 patients have inappropriate DOAC prescriptions [9–12]. Common prescribing issues include failure to adjust medication dosing appropriately for kidney or liver disease, failure to recognize potential drug-drug interactions, and failure to adjust dosing for different clinical indications as appropriate. When DOACs are mis-dosed, patients are at markedly increased risk for costly and potentially deadly bleeding or thrombotic/stroke complications.

To ensure the safe use of high-risk medications, many health systems are attempting EHR-guided population health management programs. One promising method is the use of dashboards, such as the one created by a small team of pharmacists and programmers at the United States Department of Veterans Affairs (VA) health system [13, 14]. This DOAC Dashboard assists anticoagulation pharmacists by identifying every VA patient who is prescribed a DOAC and performs an asynchronous screen from a pre-defined set of alerts for potentially risky prescribing. The tool then allows a pharmacist to click on an individual patient record, review the details of their DOAC prescribing and reason for an alert, and take an action. The DOAC Dashboard’s software interface was made available to all VA pharmacists in 2017. However, the decision to use or not use the DOAC Dashboard (site-level adoption) was left up to individual VA sites/clinics. Support for implementation was provided by the DOAC Dashboard programmer and a small team of experienced users through a nation-wide list-serve. Currently, the DOAC Dashboard is now in regular use by almost all VA anticoagulation pharmacists nation-wide, but with varying frequency of use and models for how it is incorporated into clinical workflow.

The implementation successes and failures of the DOAC Dashboard have not been clearly evaluated. Furthermore, many technological solutions in healthcare have failed to address operational and social barriers and the potential replicability of the digital tool’s implementation in sites other than VA are totally unknown. Therefore, we set out to discover the negative and positive determinants (barriers and facilitators) to effective use of the DOAC Dashboard for VA users as well as the determinants among potential non-VA users in a quality improvement initiative in the state of Michigan, USA [14]. Our goal was to use the VA experience to inform effective Dashboard introduction in a diverse set of hospital systems. We used theory-guided determinants interviews of current VA users of the dashboard and potential users in different health systems to identify promising implementation strategies.

## Methods

### Settings and participants

This project leverages two health care contexts. The first is the United States VA health system. The VA is the largest vertically and horizontally integrated health system in America [15]. Serving over 9 million veterans, it offers services at over 150 medical centers and over 1000 outpatient clinics. At the time of the interviews, the DOAC Dashboard had been available to all VA pharmacists for up to 3 years. Most participants from this setting were specialist anticoagulation pharmacists, which matches how anticoagulation care is provided in the VA health system. We also interviewed pharmacy technicians, and clinic managers who work in ambulatory anticoagulation clinics as well as the programmer who developed the VA DOAC Dashboard. Participants were identified by clinic managers and invited to participate through e-mail communication.

The second setting includes four distinct health systems that participate in the Michigan Anticoagulation Quality Improvement Initiative (MAQI^2^). Participating centers include both university-affiliated and independent centers located in urban and suburban regions of Michigan, USA. Each center has an anticoagulation clinic staffed by nurses and/or pharmacists operating under physician leadership. The participants from this setting came from a wider variety of professional backgrounds, including physician champions and medical directors, nurses and pharmacists who work in the ambulatory anticoagulation clinics, and anticoagulation clinic managers. This matches the range of caregivers who provide anticoagulation care in the MAQI^2^ sites. Most of the participants are active members of the MAQI^2^ consortium or were identified as important stakeholders by the MAQI^2^ leaders for each site. Development of a non-VA DOAC Dashboard was planned for MAQI^2^ sites at the time of the interviews.

In addition to the differences in clinical settings between the VA and MAQI^2^ sites, another important distinction is that the VA sites all had access to the DOAC Dashboard (and most were experienced users) at the time of the interviews while the MAQI^2^ sites had not yet implemented their dashboard.

### Implementation intervention development

For the larger intervention project, we are following a 7-step process to develop our implementation intervention (Table 1). This approach is similar to implementation mapping as described by Fernandez et al. and utilized recently by Klaiman et al. [16, 17] This manuscript describes steps 3–6 of the process (interviews, analysis, implementation strategy selection, and stakeholder feedback), which focus on understanding the determinants of effective implementation and guide implementation intervention development. Additional methodological details are available in the online Additional file 1 supplemental appendix. Steps 1 and 2, creating the team and identifying the intervention, preceded this work. The team was developed based on clinical and quality improvement experience related to anticoagulation care or electronic health record tool development. Many team members (physician, pharmacist, information technology programmer) has previously worked together on related projects. Other team members (project manager, qualitative expert) have not previously worked on a project in this clinical area.

**Table 1:** Implementation approach to dashboard development in MAQI^2^

Step	Details
1 – Form implementation team	We formed a MAQI^2^ implementation team consisting of a physician with expertise in anticoagulation and thrombosis, stakeholders from the anticoagulation clinic, a project manager, an EHR programmer, and staff.
2 – Identify the implementation intervention	Our goal is to help anticoagulation pharmacist/nurse staff to identify inappropriate DOAC dosing and teach them how to contact prescribers with suggested corrections to dosing. Then, we will implement the DOAC Dashboard within each health system’s EHR system, train the anticoagulation staff on the use of the DOAC Dashboard, and develop clinic policies that encourage regular use the DOAC Dashboard to identify inappropriate DOAC prescriptions and correct inappropriate DOAC prescriptions.
3 – Stakeholder interviews (see methods)	To understand what was and was not effective in a previous implementation, we conducted semi-structured interviews with key stakeholders (*n*=28) at 22 VA sites selected for having varied levels of DOAC Dashboard use. These interviews assessed for key determinants of successful implementation, focusing on how implementation was achieved, and strategies used by sites to implement and encourage effective use of the Dashboard to guide adoption in MAQI^2^. Then, to understand situations where MAQI^2^ may differ from VA, we also conducted semi-structured interviews with key stakeholders (*n*=13) at the participating MAQI^2^ centers. These formative interviews assessed for key anticipated determinants of successful implementation. They jointly focused on information pertinent to the DOAC Dashboard development process and to anticipated barriers and facilitators (negative and positive determinants) for the upcoming implementation work.
4 – Rapid qualitative analysis (see results and Table 3)	We performed rapid qualitative analysis to identify the key determinants of implementation success. Guided by the Consolidated Framework for Implementation Research and Technology Acceptance Model, we identified the most common and impactful determinants from both the VA and MAQI^2^ centers. We also identified suggested implementation strategies from the VA interviews.
5 – Select implementation strategies (see results and Table 4)	We selected potential implementation strategies for MAQI^2^ from both the items identified in the VA interviews, guided by the Expert Recommendations for Implementing Change project. These were priopritized based on the importance of their targeted determinant and perceived feasibility.
6 – Stakeholder feedback on implementation strategies (see results and Table 4)	We gathered stakeholder feedback from MAQI^2^ site leaders and anticoagulation staff about the feasibility and acceptability of individual implementation strategies. Following this, we developed a final implementation intervention plan.
7 - Evaluation	We will evaluate the success of our implementation using the Reach, Effectiveness, Adoption, Implementation, Maintenance framework [18]. We will leverage the existing MAQI^2^ registry for patient-level data and will perform semi-structured interviews with anticoagulation clinic staff and other stakeholders to assess success of implementation.

### Data collection (Step 3)

Our semi-structured interview guides (both for VA and MAQI^2^) were developed using pre-specified constructs from the Consolidated Framework for Implementation Research (CFIR) in addition to the Technology Acceptance Model (TAM) framework (Additional file 1 supplemental appendix) [19, 20]. The implementation team (GDB, ES, AS, JS) reviewed sample interview questions from cfirguide.org and published literature using TAM. Questions that were anticipated to be relevant to this project were adapted. The overall interview guide was then tested with two preliminary interviewees and edits were made to improve flow and clarity. VA sites were selected based on their level of DOAC Dashboard use (high, moderate, low/none) as calculated by the number of days per month with one or more pharmacists accessing the DOAC Dashboard. We also selected key VA sites where DOAC Dashboard use had changed significantly between 2017 and 2019 (e.g., high-to-low) to assess what specific determinants influenced the change in usage. All four MAQI^2^ sites who currently manage DOAC patients were interviewed. We identified participants at each site (VA and MAQI^2^) by asking managers to identify key front-line clinical staff.

Interviews were conducted by trained research staff (ES and AR). All participants provided verbal consent to participate and for the interviews to be recorded. Recordings were transcribed and anonymized by removing participant and site names. The interviewees collected notes during and after each interview, following the rapid qualitative analysis approach.

### Rapid qualitative analysis (step 4)

We undertook a rapid qualitative analytic approach that incorporated elements of a template analysis by using pre-existing codes from CFIR and TAM [21–23]. The three qualitative researchers (ES, AR, LT) reviewed notes taken during the interviews as well as the transcribed interviews to identify relevant themes. These were done using both pre-defined codes related to individual CFIR and TAM constructs as well as any newly emergent themes from the interviews.

### Selection of implementation strategies (step 5)

A table of key determinants identified from the interviews was created and organized according to frequency and importance, as determined by the implementation team through reviews of the interview transcripts (counting the number of coded themes) and group discussion until consensus was reached about importance. Following this prioritization activity, implementation strategies from the Expert Recommendations for Implementation Change (ERIC) project were reviewed and potential strategies were selected by the implementation team to match each prioritized determinant [24]. Additional implementation strategies suggested in the stakeholder interviews were also included.

### Stakeholder feedback on implementation strategies (step 6)

The list of prioritized determinants identified from the interviews along with suggested implementation strategies were shared with key MAQI^2^ stakeholders. Prioritization was determined by the team members based on the frequency and importance (determined in step 5) and the feasibility of paired implementation strategies selected and adapted from the ERIC project list [24]. During this session, two team members (ES and GDB) presented findings from the interviews and analysis along with a list of potential implementation strategies. Stakeholders from each MAQI^2^ site provided feedback as to the feasibility and prioritization for individual strategies.

This project was reviewed and approved by the institutional review boards of both the University of Michigan and the Ann Arbor VA.

## Results

### VA interview findings

Interviews were conducted with 32 stakeholders across 22 VA sites (Table 2). Interviews lasted an average of 38 min (range 22–61 min).

**Table 2:** Characteristics of the interviewees

Location	Dashboard use level	Number of sites	Number of interviewees	Interviewee roles
Veterans Affairs	High	19	29	Pharmacist (19), Pharmacy technician (2), Manager (7), Pharmacy resident (1)
	Moderate	2	2	Pharmacist (1), Manager (1)
	Low	1	1	Pharmacist (1)
MAQI^2^	Pre-implementation	4	13	Nurse (3), Pharmacist (4), Nurse Manager (2), Pharmacy Manager (1), Medical Director (3)

Level of Dashboard use (Veterans Affairs sites) was assessed at time of the interview or as of June 2020 if interview was conducted after that date

Five key determinants of implementation success were identified during rapid qualitative analysis of the VA transcripts. These included (1) clinician authority and autonomy; (2) clinician self-identity and job satisfaction; (3) documentation, communication, and administrative needs; (4) staffing and work schedule; and (5) technology integration (Table 3).

**Table 3:** Determinants of implementation success and associated implementation strategies

Determinants of implementation success	Additional details	Illustrative quote – experienced sites (VA)	Illustrative quote – anticipating sites (MAQI^**2**^)
Clinician authority and autonomy	Desire for staff and clinics to control/personalize their own workflow and integration of the DOAC Dashboard	“Like managing their workflow and work day so that they can incorporate it into that instead of things being maybe ‘dumped’ into their queue. So right now we’re working on, my clinical tech, our clinical tech and I are actually working on a process that we think is going to help with that.” Site H4, Study ID 173	“My concern is how would we put this in our daily workflow? Of course. That’s the biggest question I think probably everybody has.” Site 2, Study ID 4
	Concern that some centers will not allow pharmacists or nurses to make evidence-based medication changes	“I would like to see a greater change in you know, I wish that our interventions that we recommend, that more of those recommendations were taken.” Site G1, Study ID 112	“The biggest thing I can think of is physician buy-in. So, what if Doctor Smith doesn’t want somebody else touching his patient? Like, how do we protect ourselves there so we are not putting a pharmacist in a situation?” Site 3, Study ID 9
Clinician self-identity and job satisfaction	Fear that a computer program will be replacing the work of anticoagulation clinic staff and contribute to low job satisfaction	“I have heard from other facilities that their anticoagulation teams have been very reluctant to fully rely on the dashboard. I think there is a concern of losing workload credit... I think there’s also a concern or a lack of trust in the tool” Site I2, Study ID 4	n/a
Documentation, communication, and administrative needs	Concerns about how best to communicate with prescribing clinicians (e.g., physicians) and documentation burdens for a large number of patients	“So, sometimes the dosing adjustment, sometimes the indication, that's a barrier that now we have to communicate with outside provider because our providers simply likes to rewrite whatever is written outside, they're going to continue.” Site C1, Study ID 123	“Unless we get this standing protocol with a group of physicians, we can’t just act on their patients without reaching out to them first. How is reaching out to them going to be taken – how is that going to be taken? Are we going to be stepping on their toes, or they're wondering why we're reaching out to them?” Site 2, Study ID 4
	Current staff performance measures often do not include DOAC Dashboard related activities. Working with this tool might limit the ability to achieve other performance measures and revenue	“Our immediate supervisor probably knows that it’s out there and you know she may encourage us to use that for identifying our patients who are, you know how many months out or whatever, but use, no, it’s not, they don’t use it like as a performance measure or something that like” Site E2, Study ID 136	“But, where I met the biggest resistance is, why can't we charge even a small fee? Why is there no charge associated with this? Why is this something we can’t bill? They really want something billable, and something that – and really, even if we did bill them based on pharmacist charge codes, and we brought them in to see us, we would not make what it costs to have my salary.” Site 2, Study ID 4 “Right now, the anticoag clinic in its own silo is a money loser. If cardiology absorbs it, it makes their budget look bad. And for some reason, people are not seeing the bigger picture where it’s the same money coming out of the same system and it doesn’t matter whose budget it belongs to. So, that’s another barrier.” Site 1, Study ID 3
Staffing and work scheduling	Many anticoagulation clinics are busy managing warfarin-treated patients and have concerns about the extra workload of managing the DOAC Dashboard	“Well, I think a couple of barriers, one is just like being a little bit overwhelmed by the amount of alerts that we saw and thinking to ourselves, like how are we going to get this down, how are we going to keep this up on a regular basis, and then also too, I mean there were many patients that we had just never seen before.” Site I2, Study ID 4	“ … we already kind of feel like we're busting at the seams a little bit, like we’re busy 100% of the time – and that is true. But again, I can't see not pursuing something that has the potential to allow us to do a better job at what we’re currently supposed to be doing and are failing at. ‘Failing’ is maybe a harsh word, but it's true. We're not following up with every patient the way our program is designed to do. We've lost the ability to do that, and if this has the ability to bring us back to that, it increases maybe the amount of patients we need to look at in a given day, but it also increases the rate with which we discover errors that are there. We know they're there; it's just how we find them. We're not finding them now.” Site 3, Study ID 6
Integration with existing information systems	Concerns about sufficient IT resources and priority to implement	n/a	“The biggest [system barrier] is the priorities of our IT department with rolling out Epic without looking at what’s clinically more useful to us and so, right now, this tool is amazing.” Site 1 Study ID 3 [Responding to perceived organizational hurdles] “Probably the time it will take for our Epic team to figure out a way to implement in our system. Things tend to take a long time. And I believe we have already gotten approval so that approval has already been done. Yeah, so, it’s probably just a matter of how long it will take for them to get this completed.” Site 4, Study ID 11
	Uncertainty around accuracy of the DOAC Dashboard	“You kind of had to learn to trust the dashboard versus to scheduling a phone call or scheduling labs and then making sure that they’re going to go to labs or scheduling, make sure we follow up on them, whereas with this, we have to trust that the dashboard will let us know when there’s another problem that comes up.” Site D1, Study ID 5 “They certainly appreciated some of the same things, that they would be alerted to immediate abnormalities and other things but they were also concerned that they wouldn’t find bleeding events or hospitalizations or upcoming procedures or other things that we’re able to jump in and manage if they weren’t following up with patients as frequently.” Site I1, study ID 176	“I could see someone who is very critical of this type of scoring tool because you want to make sure that all of the boxes are completely accurate for the time that you're looking at the patient … And make sure to go back and double check and confirm that these patients are actively on these medications and their weights are appropriate and their creatinine are appropriate. So, source of information is going to be critical in terms of legitimizing this. They can get very credible. We want to make sure that all of these extracted pieces of information are 100% sound before facilitating a transition.” Site 3, Study ID 7
	Challenge using the DOAC Dashboard if it takes too long to load or does not integrate into existing computer system and workflow	“It is so slow, I mean that is number one. I know that’s probably not the answer you're looking for, but it is so slow.” Site E1, Study ID 53 “My anticipation is that no, we don’t intend to probably use it at any point in the near future unless there’s some other pressure to do so. With upcoming changes in pharmacy software, changes how we’re moving to Cerner, having great concerns at that juncture about tracking patients and keeping our follow-up with patients intact. I don’t suspect any further changes are probably going to be adopted.” Site 1 Study ID 176	“I can give you the example of seven years ago when [the EMR] went live and the users here had been working in a different electronic medical record before and a different software program that supported them specifically within the anticoagulation realm, and then those two things got rolled together into Epic, totally new system, and the day that Epic went live is the day that they found out that their patient records did not transfer from one to the other. So, I mean, it was a complete true disaster of knowing how to use this.” Site 3, Study ID 6

Clinician authority and autonomy were commonly identified determinants of implementation success at VA sites. Specifically, staff expressed a strong desire to control their own workflow and identify ways for the DOAC Dashboard to fit into their pre-existing workflow. Stakeholder interviewees also expressed concerns about the level of autonomy they would have for making guideline recommended DOAC dose changes when the DOAC Dashboard alerted them to an unsafe prescription. This was particularly troubling for some pharmacists when they had to alert a prescribing clinician rather than make the change themselves. Once they were aware of a DOAC dosing error, they found a lack of autonomy limited their ability to enact meaningful changes if the prescribing clinician did not promptly respond to their messages. They noted that without the knowledge of a DOAC prescribing error identified by the DOAC Dashboard, they would not feel an obligation to “fix” the prescribing error. Importantly, the issue around autonomy was not a direct result of the DOAC Dashboard but rather the variation in practice authority and autonomy given to pharmacists or nurses across the USA.

Clinician self-identity and job satisfaction was closely linked to how robustly they integrated the DOAC Dashboard into their practice. Some pharmacists expressed a concern that the computer is replacing their clinical judgement or justification for their work. Furthermore, many VA pharmacists who were used to seeing patients face-to-face feared the loss of direct patient care if they no longer had scheduled visits and instead relied only on the DOAC Dashboard to identify potential dosing errors.

Documentation and work performance barriers were commonly cited by many VA stakeholder interviewees. These include difficulties communicating with primary care providers and specialists both within and outside the VA health system, a problem that is not unique to the DOAC Dashboard itself and often requires additional staff time to complete. They also expressed concern that staff performance measures may not include DOAC Dashboard work if there is not sufficient documentation to account for the time spent reviewing charts and communicating with other clinicians or the patient.

Having sufficient staff and scheduled time to work with the DOAC Dashboard was a common determinant of implementation success. Stakeholder interviewees who felt the DOAC Dashboard was highly successful tended to describe a workflow that included dedicated staff and time to review the dashboard and make clinically appropriate changes. This included interviewees at sites that developed pure “dashboard clinics,” days in which the pharmacists would work primarily on addressing flags. This would allow them to extend the length between visits for patients who did not have flags. In distinction, stakeholder interviewees that expressed difficulty using the dashboard often worked at sites where the DOAC Dashboard was added to existing workflow. This was particularly true when sites first began using the DOAC Dashboard because of the large number of alerts they encountered. Over time, this number was reduced, and interviewees reported a manageable “steady state.”

Lastly, additional concerns about integration with existing information systems were cited by some VA interviewees. Two major areas were highlighted, including uncertainty around accuracy of the tool and the speed with which it loads and can be used. Some pharmacists expressed a lack of trust in the dashboard, finding it not always accurate and missing individual patients for whom the dashboard did not show an alert. This was seen as a barrier for clinicians used to reviewing every patient that they followed on a regular basis.

### Comparison of VA and MAQI^2^ interview findings

Thirteen stakeholders at four MAQI^2^ sites participated in interviews (Table 2). These interviews lasted an average of 42 min (range 27–50).

Four of the five determinants from the VA interviews were identified in the MAQI^2^ interviews. These included (1) clinician authority and autonomy; (2) documentation, communication, and administrative needs; (3) staffing and work schedule; and (4) integration with existing information systems (Table 3). Clinician self-identity and job satisfaction were not identified in the MAQI^2^ interviews. Opinions on documentation and administrative needs and staffing and work schedule identified by MAQI^2^ interviewees were very aligned with those of their VA counterparts. Clinician authority and autonomy and technology were notable differences, as detailed below.

Regarding authority and autonomy, the MAQI^2^ stakeholders identified that regulatory barriers would need to be addressed in ways that were not identified in the VA interviews (Table 3). Specifically, the nurses and pharmacists in the MAQI^2^ centers work under collaborative agreements with specific physician groups at their hospitals. Currently, very few patients treated with DOACs are individually referred to the anticoagulation clinic for nurse and/or pharmacist monitoring. To maximize impact, the MAQI^2^ DOAC Dashboard is designed to monitor all DOAC-treated patients across a health system or who are managed by large groups of physician organizations, not just those who were specifically referred to the anticoagulation clinic for monitoring. Therefore, many of those agreements will need to be updated so that all DOAC-treated patients within a health system can be managed by the nurses and/or pharmacists in the anticoagulation clinic without individual referral. Furthermore, the DOAC Dashboard can only be used for patients who are being managed by physicians with existing anticoagulation clinic practice agreements and cannot be used to monitor patients managed by other physician groups within the hospital or health system. This is notably different than the VA system, which as a federal agency of the US government operates under very different rules and regulations from non-federal health systems. Non-VA nurses and pharmacists are required to operate under individual state rules and regulations as well as often working with independent, self-employed physician groups.

Technological concerns were even more salient in the MAQI^2^ interviews than in the VA. In particular, concerns about reliability/trust in the accuracy of the tool and the speed with which the tool loaded (Table 3) were very prevalent. Interviewees did not identify resources or time needed to initially implement the DOAC Dashboard as a major barrier. Unlike in VA, the MAQI^2^ interviews frequently cited concerns with how limited access to dedicated information technology staff members who are ultimately responsible for any changes to the electronic health record system. Specifically, they felt that the limited access to these professionals would harm the adoption of the dashboard and how implementing the dashboard into the electronic health record might burden those information technology staff from other immediate needs. They also frequently cited concerns about a lack of access to medical records from outside their health system, a barrier not frequently noted by VA interviewees due to availability of a nation-wide VA EHR records.

### Implementation strategy selection and stakeholder feedback

Based on the findings from both the VA and MAQI^2^ interviews, our implementation team identified a set of strategies aimed at addressing key determinants of successful implementation (Table 4). These strategies were prioritized based on the relative frequency of their targeted determinant and feasibility. These were reviewed and endorsed by the MAQI^2^ stakeholder focus group with broad and enthusiastic support regarding feasibility and impact.

**Table 4:** Strategies for MAQI^2^ implementation endorsed by stakeholder group

Implementation strategy	Additional details	Targeted determinant of implementation success
Create new teams	These customized teams (including pharmacists, nurses, technologists, and/or administrative assistants) at each center will tailor workflow that best meets the needs of each anticoagulation clinic staff and culture	Clinician authority and autonomy AND Staffing and scheduling
Create new guidelines, update policies, and revise professional roles	These updates will focus on clinic-lead medication changes for unsafe DOAC dosing that minimize reliance on referring physicians	Clinician authority and autonomy
Develop note and communication templates	Ensure these templates are easy to use for communicating with physician colleagues within and outside each MAQI^2^ hospital	Documentation, administrative needs, and performance evaluation
Capture and share local knowledge	Share notes developed at other sites, especially early adopter sites. Leverage the learning collaborative to share these tools.	Documentation, administrative needs, and performance evaluation
Develop and organize a quality monitoring system	Build into EHR a means for monitoring DOAC Dashboard use and impact to quantify staff work.	Documentation, administrative needs, and performance evaluation
Alter performance measures	Engage clinic leadership to alter staff performance measures that include DOAC Dashboard use	Documentation, administrative needs, and performance evaluation
Access new funding	Use additional funding to support additional team members to work with or support DOAC Dashboard use. This will require robust metrics to demonstrate return on investment.	Staffing and scheduling
Stage scale up	To address the initial volume of alerts, temporary staff or a planned role out over time can be used to reduce burden	Staffing and scheduling
Provide and prioritize local technical assistance	Alert information technology teams months in advance of required implementation needs to allow for appropriate prioritization	Technology integration
Centralize technical assistance	A single developer with provide technical assistance to all MAQI^2^ sites	Technology integration
Early adopter demonstration	Use data from the early adopter sites to demonstrate the accuracy of the DOAC Dashboard	Technology integration
Trialability and customization	Allow sites to try out the DOAC Dashboard and make customizations (e.g., which patients are included, thresholds for alerts).	Technology integration
User-centered design approach	Follow a user-centered design approach to initial development of the DOAC Dashboard based on early adopter site feedback. Improve layout and load time of the tool. Build the tool directly within EHR to maximize workflow integration and security.	Technology integration

To address concerns about control over each individual’s personal workflow to use the DOAC Dashboard effectively (authority and autonomy), the MAQI^2^ stakeholders agreed that multi-disciplinary teams should pilot and revise workflows as needed. To accomplish this work, new clinical teams will need to be created, leveraging pharmacists, nurses, pharmacy technicians, and administrative assistants. Furthermore, sites agreed to create clinic-led medication change guidelines and to update institutional policies that allow anticoagulation clinic staff to change unsafe DOAC dosing or use.

Within the MAQI^2^ sites, technological issues, specifically fears of long loading times and the tool’s accuracy, were the most commonly cited potential barrier to a successful use of the DOAC Dashboard. To address these concerns, implementing sites decided to work with a central IT programmer to promote local technical expertise and assistance as well as virtual visits to early adopter sites. Rapid cycling to make changes when clinical guidance evolves (e.g., changes in approved DOAC indications) is a high priority for the MAQI^2^ implementation team. Finally, trialability with the DOAC Dashboard will be encouraged to build trust in the digital tool at each MAQI^2^ site. Specifically, after technical implementation, sites will have an opportunity to trial use of the DOAC Dashboard before developing clinical protocols. During this trial, the MAQI^2^ programmer will be available for technical support and all MAQI^2^ sites will be encouraged to provide peer support through regular monthly conference calls.

While many implementation projects draw from a robust list of implementation strategies targeted to specific determinants, this project had to identify strategies that were unique to technology-based implementation. The central IT programmer is familiar with the design of the dashboard and will assist both local clinical and IT partners with technological implementation. He is also able to identify technical problems at one site and quickly disseminate potential solutions at the other sites. Unlike many implementation projects that do not rely heavily on IT tools, this project requires unique skills that rarely are found within clinical champions. Therefore, it is paramount that the IT and clinical teams work closely together to achieve successful implementation.

## Discussion

Through interviews with 45 diverse stakeholders, we identified five key determinants of implementation success for an EHR-based tool for safe medication use. These include clinician authority and autonomy, clinician self-identity and job satisfaction, documentation/communication and administrative issues, staffing and scheduling, and integration with existing information systems. These concerns were similar for the VA providers who have been using the dashboard as for the MAQI^2^ providers who will be adopting the tool. For each set of important implementation determinants, we have also identified key implementation strategies that our stakeholder group feel are feasible and impactful. Many of these have already been successfully used at some or all MAQI^2^ sites.

### Differences between VA and MAQI^2^ interviews

Two important distinctions between the VA and MAQI^2^ interviews warrant discussion. First, clinician self-identity and job satisfaction were not identified as a concern in the MAQI^2^ interviews. Though this issue may emerge after the MAQI^2^ complete implementation, it may reflect important differences between the VA and MAQI^2^ anticoagulation clinics and their staff. In the VA, most anticoagulation clinic staff are pharmacists who practice at the top of their license. While phone-based contact with patients is used, face-to-face anticoagulation care is quite common. Additionally, many anticoagulation clinics in the VA serve not only to adjust warfarin dose based on monthly labs, but they also support perioperative management and assist with other related care practices. In contrast, at the four MAQI^2^ anticoagulation clinics, the clinicians are predominantly nurses, all care is phone-based, and patients prescribed DOAC medications are rarely referred for care to the anticoagulation clinic. As a result, the clinical training and work tasks most common in each setting may dictate the importance (or presence) of certain implementation determinants for new programs that aim to fundamentally change the way clinical care is delivered.

The second important difference was that only MAQI^2^ interviewees expressed strong concerns with the availability of sufficient information technology (IT) resources and ability to prioritize this digital tool project. Many MAQI^2^ interviewees shared concerns with prior EHR implementation efforts that were not accomplished as quickly as desired. This likely reflects that the DOAC Dashboard had not yet been implemented at these centers when the interviews occurred, while all VA interviewees had used the VA Dashboard. This shows how much the timing of an assessment is an important consideration for comparing experienced to novice users or sites in any implementation project. Concerns about IT programmer workload and prioritization for IT implementation have been identified as important barriers in similar work across other clinical domains [25]. While decentralized technology-associated solutions (e.g., SMART on FHIR application programming interface) have been proposed to reduce the programmer burden at individual sites, these tools are not yet widely implemented at many health centers. Furthermore, institutional leaders (e.g., chief medical information officers) have variable desire to control the logic and flow of information when digital tools are implemented outside of (but interfacing with) their EHR system.

### Impact beyond the DOAC Dashboard

Our work has proven very helpful as we prepare to implement the DOAC Dashboard within the MAQI^2^ centers. We also hope that this can become a generalized approach to high-stakes technological implementations in other spheres of medicine. While much work in implementation science has focused on the use of non-electronic interventions, there has been less focus understanding how to effectively operationalize and use digital tools as implementation strategies aimed at promoting safe and effective medication management [26, 27]. Emblematic of this, commonly used determinant frameworks include very few items specific to technology [18, 19, 28]. Specifically, CFIR does not include information technology/systems as a construct in its current form while the Tailored Implementation of Chronic Diseases checklist has a single item for “Information System.” In contrast, emerging health information technology frameworks are being developed to detail the numerous factors that influence successful EHR- and technology-based implementation within health care delivery [29, 30]. Integrating these frameworks/checklists is an important area of future research.

Similarly, compiled lists of implementation strategies do not include a diverse array of technology-associated options [24]. Nonetheless, healthcare delivery in the twenty-first century is becoming increasingly dependent on technology with the rapid adoption of EHRs in both the hospital- and ambulatory-care settings. Our project, which aims to leverage EHR technology to facilitate safe anticoagulant prescribing, is just one example of how clinicians can leverage technology to ensure evidence-based practices are followed. Notably, three distinct etiologies of implementation determinants were identified from our stakeholder interviews: (1) variation in external environments/policies that differentially limit pharmacist or nurse autonomy, (2) direct effect of the digital tool on how clinicians view their decision-making capacity, and (3) interaction between clinician workflow/environment and the digital tool.

The second issue parallels commonly cited barriers by many physicians who see guidelines as limiting individual clinical judgement in favor of “cookbook medicine.” [31, 32] Future work should explore how technology serves both as a determinant of implementation success (i.e., resources required to install and use the DOAC Dashboard) and how it can be used as a strategy to overcome key barriers (i.e., use a DOAC Dashboard to identify overlooked prescribing errors by non-experts). This work will likely require that technology-associated efforts are described in detail so that the individual elements can be tested for impact on evidence-based practice.

Our implementation intervention development has important limitations that must be acknowledged. First, most of our stakeholder interviews were conducted with VA clinicians who were identified by clinic managers. It is not yet clear how well these experiences translate to the non-VA healthcare setting. We plan to conduct post-implementation interviews with MAQI^2^ stakeholders to assess for any important differences. We also cannot exclude a potential for selection bias in our participants. However, the large number of interviews and intentional inclusion of sites with varied DOAC Dashboard use should minimize this impact. Second, while the MAQI^2^ DOAC Dashboard was developed to closely mirror the VA DOAC Dashboard, it is inherently different given the different EHR systems. However, the underlying elements (patient identification, rules for medication alerts) are the same and therefore should not meaningfully impact the implementation or evaluation. Third, while we often included multiple interviewees from a single site, some sites were only able to identify a single stakeholder to participate in the interview. Finally, the findings from this study may not be applicable to the development and implementation of other EHR-based tools for other medications or clinical care delivery. For instance, not all medications have such complex dosing regimens (e.g., anti-hypertensive medications) or are titrated based on factors not commonly collected within the electronic health record (e.g., anxiety symptom control).

## Conclusion

In summary, we have identified several key operational and social barriers to EHR tool implementation within the health care system. Using a theory-informed process, we have developed a set of implementation interventions aimed at improving evidence-based anticoagulant prescribing using an EHR-based population health tool. Future work will evaluate this implementation intervention in a diverse set of health systems. Additional work to better incorporate granular technology issues in key implementation determinants frameworks and implementation strategies lists will greatly benefit teams looking to leverage this powerful and rapidly expanding healthcare tool.

## Supplementary Information

**Additional file 1.** Additional Methodological Detail.

## Data Availability

The datasets generated and/or analyzed during the current study are not publicly available due to the Department of Veterans Affairs’ regulatory compliance.

## Abbreviations

CFIR: Consolidated Framework for Implementation Research
DOAC: Direct oral anticoagulant
EHR: Electronic health record
IT: Information technology
MAQI^2^: Michigan Anticoagulation Quality Improvement Initiative
TAM: Technology acceptance model
VA: Veterans affairs

## References

[1] Austin J, Barras M, Sullivan C (2020). Interventions designed to improve the safety and quality of therapeutic anticoagulation in an inpatient electronic medical record. Int J Med Inform.

[2] Allen AL, Lucas J, Parra D, Spoutz P, Kibert JL, Ragheb B, et al. Shifting the paradigm: a population health approach to the management of direct oral anticoagulants. J Am Heart Assoc. 2021:e022758 Available from: http://www.ncbi.nlm.nih.gov/pubmed/34796718. 10.1161/JAHA.121.022758 PMC9075229 34796718

[3] Bejjanki H, Mramba LK, Beal SG, Radhakrishnan N, Bishnoi R, Shah C (2018). The role of a best practice alert in the electronic medical record in reducing repetitive lab tests. Clin Outcomes Res..

[4] Heidbuchel H, Verhamme P, Alings M, Antz M, Diener HC, Hacke W (2015). Updated European Heart Rhythm Association Practical Guide on the use of non-Vitamin K antagonist anticoagulants in patients with non-valvular atrial fibrillation. Europace..

[5] Hindricks G, Potpara T, Dagres N, Arbelo E, Bax JJ, Blomström-Lundqvist C (2021). 2020 ESC Guidelines for the diagnosis and management of atrial fibrillation developed in collaboration with the European Association for Cardio-Thoracic Surgery (EACTS). Eur Heart J.

[6] January CT, Wann LS, Calkins H, Chen LY, Cigarroa JE, Cleveland JC (2019). AHA/ACC/HRS Focused update of the 2014 AHA/ACC/HRS Guideline for the management of patients with atrial fibrillation: a report of the American College of Cardiology/American Heart Association Task Force on Clinical Practice Guidelines and the Heart R. J Am Coll Cardiol.

[7] Ortel TL, Neumann I, Ageno W, Beyth R, Clark NP, Cuker A (2020). American Society of Hematology 2020 guidelines for management of venous thromboembolism: treatment of deep vein thrombosis and pulmonary embolism. Blood Adv.

[8] Renner E, Barnes GD (2020). Antithrombotic management of venous thromboembolism: JACC focus seminar. J Am Coll Cardiol..

[9] Camm AJ, Cools F, Virdone S, Bassand JP, Fitzmaurice DA, Arthur Fox KA (2020). Mortality in patients with atrial fibrillation receiving nonrecommended doses of direct oral anticoagulants. J Am Coll Cardiol..

[10] Gregory H, Cantley M, Hall GA, Matuskowitz AJ, Weant KA. Incidence of anticoagulation medication prescribing errors in patients discharged from the emergency department. J Am Coll Clin Pharm. 2020:1–6.

[11] Steinberg BA, Shrader P, Thomas L, Ansell J, Fonarow GC, Gersh BJ (2016). Off-label dosing of non-vitamin K antagonist oral anticoagulants and adverse outcomes: the ORBIT-AF II registry. J Am Coll Cardiol.

[12] Dawson T, Decamillo D, Kline-Rogers E, Haymart B, Froehlich JB, Barnes GD (2018). Correcting inappropriate prescribing of direct oral anticoagulants: a population health approach. Res Pr Thromb Haemost..

[13] Rossier C, Spoutz P, Schaefer M, Allen A, Patterson ME. Working smarter, not harder: evaluating a population health approach to anticoagulation therapy management. J Thromb Thrombolysis. 2020; Available from: 10.1007/s11239-020-02341-y. 10.1007/s11239-020-02341-y 33222115

[14] Valencia D, Spoutz P, Stoppi J, Kibert JL 2nd, Allen A, Parra D, et al. Impact of a direct oral anticoagulant population management tool on anticoagulation therapy monitoring in clinical practice. Ann Pharmacother. 2019;2019(1060028019835843) Available from: https://www.ncbi.nlm.nih.gov/pubmed/30854862. 10.1177/1060028019835843 30854862

[15] About VHA - Veterans Health Administration [Internet]. [cited 2020 Jul 23]. Available from: https://www.va.gov/health/aboutvha.asp

[16] Fernandez ME, ten Hoor GA, van Lieshout S, Rodriguez SA, Beidas RS, Parcel G (2019). Implementation mapping: using intervention mapping to develop implementation strategies. Front Public Heal..

[17] Klaiman T, Silvestri JA, Srinivasan T, Szymanski S, Tran T, Oredeko F (2021). Improving prone positioning for severe acute respiratory distress syndrome during the covid-19 pandemic an implementation-mapping approach. Ann Am Thorac Soc..

[18] Flottorp SA, Oxman AD, Krause J, Musila NR, Wensing M, Godycki-Cwirko M (2013). A checklist for identifying determinants of practice: a systematic review and synthesis of frameworks and taxonomies of factors that prevent or enable improvements in healthcare professional practice. Implement Sci.

[19] Damschroder LJ, Aron DC, Keith RE, Kirsh SR, Alexander JA, Lowery JC (2009). Fostering implementation of health services research findings into practice: a consolidated framework for advancing implementation science. Implement Sci.

[20] Holden RJ, Karsh BT (2009). The technology acceptance model: its past and its future in health care. J Biomed Inf.

[21] Beebe J (2014). Rapid Qualitative Inquiry.

[22] Brooks J, McCluskey S, Turley E, King N (2015). The utility of template analysis in qualitative psychology research. Qual Res Psychol.

[23] McIlvennan CK, Morris MA, Guetterman TC, Matlock DD, Curry L (2019). Qualitative methodology in cardiovascular outcomes research: a contemporary look. Circ Cardiovasc Qual Outcomes.

[24] Powell BJ, Waltz TJ, Chinman MJ, Damschroder LJ, Smith JL, Matthieu MM (2015). A refined compilation of implementation strategies: results from the Expert Recommendations for Implementing Change (ERIC) project. Implement Sci.

[25] Gesell SB, Golden SL, Limkakeng AT, Carr CM, Matuskowitz A, Smith LM (2018). Implementation of the HEART pathway: using the consolidated framework for implementation research. Crit Pathways Cardiol A J Evidence-Based Med.

[26] Barnes GD, Misirliyan S, Kaatz S, Jackson EA, Haymart B, Kline-Rogers E (2017). Barriers and facilitators to reducing frequent laboratory testing for patients who are stable on warfarin: a mixed methods study of de-implementation in five anticoagulation clinics. Implement Sci.

[27] Greiver M, Dahrouge S, O’Brien P, Manca D, Lussier MT, Wang J (2019). Improving care for elderly patients living with polypharmacy: protocol for a pragmatic cluster randomized trial in community-based primary care practices in Canada. Implement Sci..

[28] Cane J, O’Connor D, Michie S (2012). Validation of the theoretical domains framework for use in behaviour change and implementation research. Implement Sci.

[29] Singh H, Sittig DF (2016). Measuring and improving patient safety through health information technology: the health IT safety framework. BMJ Qual Saf..

[30] Singh H, Sittig DF (2020). A sociotechnical framework for safety-related electronic health record research reporting: the SAFER reporting framework. Ann Intern Med..

[31] Cabana MD, Rand CS, Powe NR, Wu AW, Wilson MH, Abboud P-AC (1999). Why don’t physicians follow clinical practice guidelines?. JAMA.

[32] Bierbaum M, Rapport F, Arnolda G, Nic Giolla Easpaig B, Lamprell K, Hutchinson K (2020). Clinicians’ attitudes and perceived barriers and facilitators to cancer treatment clinical practice guideline adherence: a systematic review of qualitative and quantitative literature. Implement Sci. Implement Sci.

