# Correction: Implementing an electronic health record dashboard for safe anticoagulant management: learning from qualitative interviews with existing and potential users to develop an implementation process

**DOI:** 10.1186/s43058-023-00412-8

**Published:** 2023-03-13

**Authors:** Geoffrey D. Barnes, Emily Sippola, Allison Ranusch, Linda Takamine, Michael Lanham, Michael Dorsch, Anne Sales, Jeremy Sussman

**Affiliations:** 1 Frankel Cardiovascular Center, Department of Internal Medicine, University of Michigan, 2800 Plymouth Rd, B14 G214, Ann Arbor, MI 48109-2800 USA; 2 Center for Behavioral and Social Sciences in Medicine, University of Michigan, Ann Arbor, USA; 3 Institute for Healthcare Policy and Innovation, University of Michigan, Ann Arbor, USA; 4 Center for Clinical Management Research, Ann Arbor Veterans Health Affairs, Ann Arbor, USA; 5 Department of Learning Health Sciences, University of Michigan, Ann Arbor, USA; 6 College of Pharmacy, University of Michigan, Ann Arbor, USA; 7 Division of General Medicine, Department of Internal Medicine, University of Michigan, Ann Arbor, USA

**Correction: Implement Sci Commun 3, 10 (2022)**

**https://doi.org/10.1186/s43058-022-00262-w**

Following the publication of the original article [1] the authors requested to update the “Competing interests” section as follows:

“The authors declare that Anne Sales is co-Editor-in-Chief of the journal."
