# Supplementary material for: Implementing an electronic health record dashboard for safe anticoagulant management: learning from qualitative interviews with existing and potential users to develop an implementation process
Source: Implement Sci Commun. 2022 Feb 2;3:10. doi: 10.1186/s43058-022-00262-w (PMC8812192; doi:10.1186/s43058-022-00262-w)
Supplement: Supplementary file 1 — Additional file 1. Additional Methodological Detail. [file 43058_2022_262_MOESM1_ESM.pdf]

Online Supplemental Appendix: Additional Methodological Details

<u>Data Collection (Step 3)</u>
An initial guide was developed for anticoagulation staff and then a second was created by adapting for physician and non-clinical leaders. Slightly different versions of the guide were used for Veterans Health Administration (VHA) interviewees who had experience with the DOAC Dashboard than for Michigan Anticoagulation Quality Improvement Initiative (MAQI^2) subjects, where the DOAC Dashboard had not yet been implemented. The MAQI^2 guide included a brief overview of the DOAC Dashboard and a few screen shots to orient the interviewee to the tool.

For the VHA interviews, we approached anticoagulation clinic managers for permission to interview their anticoagulation clinic staff. If any interview subject suggested that we reach out to additional stakeholders (e.g., pharmacy technician, physician), we invited them to participate. For the MAQI^2 interviews, the medical director and/or clinic administrative lead were approached and invited to participate.

Due to the COVID-19 pandemic, all interviews with VHA pharmacists and anticoagulation staff occurred using phone-based conference call systems. Interviews MAQI^2 anticoagulation staff (nurses and pharmacists) and leaders (operational and physician) were conducted by phone- and web-based systems.

<u>Rapid Qualitative Analysis (Step 4)</u>
One interviewer (AR) is a VHA employee with insider knowledge about VHA-specific language, policies, and norms (insider). The other interviewer (ES) was external to the VHA system but had been working closely with the MAQI^2 sites in preparation for implementation of the DOAC Dashboard. Most interviews were conducted with both interviewers present. Both interviewers took field notes and meet weekly with two other team members (GDB and JS) and a third qualitative researcher (LT) to discuss findings. GDB and JS are both physicians, one is an expert in anticoagulation at a participating MAQI^2 site and the other is a VHA provider. To assist with better understanding from the interviews, the two physicians frequently discussed findings and emerging themes with two VHA pharmacists who initially designed and implemented the VHA DOAC Dashboard as well as the programmer leading the MAQI^2 Dashboard development.

<u>Selection of Implementation Strategies (Step 5)</u>
An initial set of strategies that addressed identified determinants (step 4) were selected based on the ERIC project list. Since early implementation work within MAQI^2 was ongoing, individual strategies were then listed according to their current status (completed, in progress, future). The implementation team reviewed all strategies and created an initial prioritization list based on perceived feasibility and impact.

DOAC Dashboard Barriers and Facilitators Exploratory Research
Discussion Guide

- 30-45 minute interviews with non-, med-, and high-use sites VA sites
- 30-45 minute interviews with MAQI clinical sites

Objective: Understand known and perceived barriers and facilitators to use of DOAC Dashboard tool within the respondent's own health system

A. Identify human facilitators which have contributed or would contribute to adoption of or resistance to the DOAC Dashboard on site
B. Identify communication strategies that influenced DOAC Dashboard adoption and use
C. Identify the logistical barriers and facilitators that can drive adoption of the DOAC Dashboard
D. Identify the forms of infrastructure that benefit or limit adoption and use of the DOAC Dashboard

NOTE: This discussion guide acts as an outline for the conversation between the respondent and the researcher. Researchers have made efforts to craft all probes that may be needed, but the interviewer may ask additional questions to meet the overarching and specific research objectives. Language and question order may be modified for the researcher's or respondent's comfort and needs and to improve the flow of the questions and conversation.

Group	Questions and Probes	Framework / Elements
All	**Introduction and Warm-up**<br>a. Moderator introduction, consent, and overall study objectives: *My name is _____, and I'm a researcher working with the University of Michigan/Ann Arbor VA. This interview is a part of a larger research study we're conducting with people who are involved in the treatment and care of patients taking anticoagulants, specifically DOACs. We are trying to understand how your work flows in caring for these patients and how a dashboard or app or online tool that evaluates patients on DOACs fits into that work flow. There are no right or wrong answers to the questions I'm going to ask today, and all of your responses and this recording are completely confidential and will be stored on a secure server without any identifying information about you or your employer or location. Do I have your permission to continue with the recording?*<br>b. Professional background<br>i. Practice setting, job title, years in this kind of setting, years in current role<br>ii. How many patients on anticoagulant therapy touched per month? In what capacity?<br>1. Record review<br>2. Clinic<br>3. Phone<br>iii. What is the reach of the anticoagulation clinic in your setting, if any?	Individual Professional Characteristics of Individuals (CFIR)

Group	Questions and Probes	Framework
All	**DOAC Dosing and Medication Errors: Current State**<br>a. Who usually spots DOAC problems in your setting (if nec: without the DOAC Dashboard tool)? What is the process for handling these errors?<br>iii. How are DOAC problems identified?<br>iv. Who first finds the problem?<br>v. How are the problems evaluated?<br>vi. How is the DOAC problem resolved?<br>vii. What tools do you have available to you to make sure that your patients on DOACs are appropriately managed?	Structural Characteristics, Networks and Communications, Culture (CFIR); Perceived ease-of-use (TAM)
All	b. In what ways does the process break down? What frustrations do you have with the current flow? Which parts take the longest time?<br>c. In what ways has it been successful?	Complexity, Compatibility, Readiness for Implementation, Knowledge and Believe about the Intervention, Planning, Reflecting and Evaluating (CFIR); Perceived ease-of-use, Perceived usefulness (TAM)
All	d. How have these processes been evolving in recent years at your site?<br>iii. Probes:<br>1. (IF MAQI) Participation with the MAQI[2] consortium<br>2. (IF VA) Use of Dashboard	Tension for Change, Reflecting and Evaluating (CFIR)
MAQI and VA non-user	a. Present respondent with DOAC DASHBOARD description and images: *This is a description of a tool that is / can be built into the electronic health record of your health system to evaluate patient records and flag any patients who are taking a DOAC and have a contraindication or dosing error (show a picture of what the Dashboard looks like). What are your initial thoughts?*<br>i. Seek to understand the following:<br>1. What benefits do you see to this tool in your current workflow?<br>2. Drawbacks?<br>ii. What questions would you have for the developer?<br>iii. Who in your workgroup would be the biggest champion of a tool like this? The biggest hurdle?	Relative Advantage, Complexity, Design Quality and Packaging, Compatibility, Champions (CFIR); Perceived ease-of-use, Perceived usefulness, Intent to use, Social Influence and Subject Norms (TAM)
MAQI and VA non-user	b. Revisit frustrations, resources, timing, unmet needs:<br>i. Which of your prior frustrations / process breakdowns would be alleviated through a tool like this?<br>c. What do you think would be more complicated or difficult if this tool were adopted in your center?<br>d. What information about this tool would be most important to the people deciding whether to use it?	Relative Advantage, Trialability; Design Quality and Packaging, Culture, Compatibility, Opinion Leaders, Champions (CFIR); Perceived ease-of-use, Perceived usefulness (TAM)

Group	Questions and Probes	Framework
VA Dashboard Users	a. Tell me about the DOAC tool you use for the patients in your health system.<br>i. When did your team start using it? When did you start using it?<br>ii. Who in your team brought it on board for use? (title, role)<br>1. How did that person explain the tool? Were they a champion for use of the tool or just a messenger?<br>iii. What was the onboarding process? (growing pains, ease of moving into workflow)<br>b. What has the overall impact of this tool been in your work and patient care?<br>i. Probe on time spent, communication with colleagues and patients, impact on patients directly<br>ii. What are the benefits you see in using this?<br>iii. Drawbacks?<br>c. What has limited your use of this tool in your current setting?<br>i. Process requirements, colleagues, red tape<br>d. What has expanded your use of the tool?	Trialability, Complexity, Compatibility, Available Resources, Champions, Reflecting and Evaluating (CFIR) Perceived ease-of-use, Perceived usefulness, Perceived behavioral control and facilitating conditions (TAM)
